# Airway compromise in infectious mononucleosis: a case report

**DOI:** 10.4076/1757-1626-2-6736

**Published:** 2009-08-13

**Authors:** Sravan Kakani

**Affiliations:** Louis Stokes Cleveland Veterans Affairs Medical Center10701 East Boulevard, Cleveland, OH, 44106USA

## Abstract

A 25-year-old Caucasian man had difficulty swallowing and shortness of breath during an episode of infectious mononucleosis. His tonsils were “kissing” and erythematous but no superimposed infection with a streptococcal organism was identified. His symptoms improved rapidly upon administration of intravenous steroids. This case demonstrates a rare and short-term complication that is well described in young adults with infectious mononucleosis. Physicians should routinely counsel their patients with infectious mononucleosis to be aware of potentially life-threatening airway obstruction in addition to splenic rupture and meningitis.

## Introduction

Infectious mononucleosis is usually a benign disease that rarely presents with symptoms of dyspnea, dysphagia, and odynophagia as a result of significant pharyngitis or tonsillitis. The differential diagnosis includes infectious causes such as diphtheria, epiglottitis, infectious mononucleosis, Ludwig’s angina, peritonsillar abscess and non-infectious causes such as angioedema, neoplasms, foreign bodies and local trauma [1]. In this case, a patient with an incidental diagnosis of mononucleosis, developed tonsillar swelling with subsequent airway compromise leading to hospital admission.

## Case presentation

A 25-year-old Caucasian male with a history of tobacco use, alcohol abuse, and gastroesophageal reflux disorder (GERD) was admitted to our hospital with a chief complaint of upper airway obstruction. He was initially seen one week prior to admission by his primary care physician for a complaint of back pain. At this visit, the patient reported recent contact with a co-worker who was recovering from mononucleosis and thus a blood smear was performed which revealed atypical lymphocytes. A subsequent Monospot test was positive for heterophile antibodies. Further blood tests for viral hepatitis were negative. He first began developing symptoms four days after diagnosis, which included fevers, nightsweats, diffuse body aches, and a sore throat. These continued to worsen in the following two days, so the patient sought attention at the urgent care clinic. Workup at this time for superimposed *streptococcus* infection was negative. The patient returned home without treatment. Symptoms worsened overnight including difficulty swallowing and handling oral secretions as well as fevers to 104°F. Two days later, the patient was short of breath with continued fevers, throat pain, and difficulty swallowing leading to one episode of blood-tinged emesis.

Medical history included GERD, anxiety, and moderate tobacco and alcohol use. He was on no medications. His family history was significant for epilepsy and hyperthyroidism. He was a student and worked as a manager of a restaurant. He drank five beers 3-4 times per week, smoked a ½ pack per day, and denied illicit drug use. The patient had no known drug allergies.

On physical exam, he was alert and awake with a flushed face and spoke with a hyponasal or raspy voice. This muffled vocal characterization is referred to as “hot potato voice” by some physicians [2]. He was in no apparent respiratory distress. Height was 72 inches and weight was 191 pounds. His vitals were T 99.3°F, P 106, R 24, BP 133/88, 96% on room air. Head and neck exam revealed tender cervical lymphadenopathy with erythematous, swollen tonsils that were in apposition with minimal white exudates. There was no evidence of inflammation in the internal auditory canal, sclera were non-injected, no stridor, no trismus, and the trachea was midline. Lung exam was clear to auscultation bilaterally. Heart sounds were regular without murmurs or clicks. The abdomen was soft, non-tender, and without hepatosplenomegaly. Extremities were without edema, clubbing, or cyanosis. Neurological exam revealed no focal deficits. Laboratory values were remarkable for a white blood cell count of 20 K/cmm with an absolute monocytosis of 2.97 K/cmm. Liver enzymes were elevated with an alkaline phosphatase of 270 U/L, AST 136 U/L, ALT 275 U/L.

**Figure 1.**
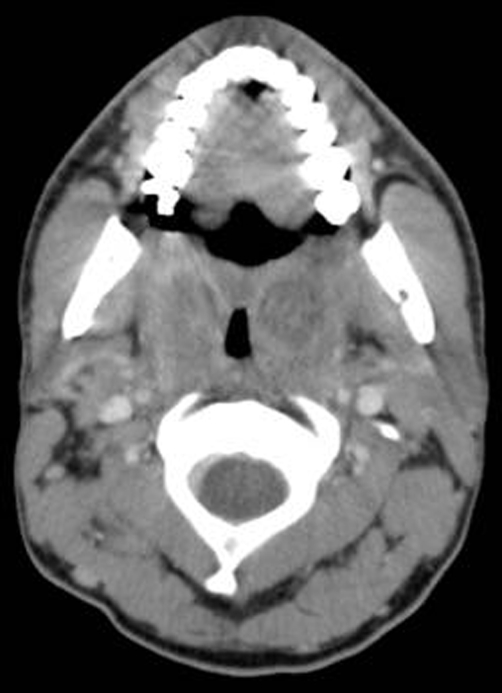
CT scan of head and neck. Note tonsillar swelling.

**Figure 2.**
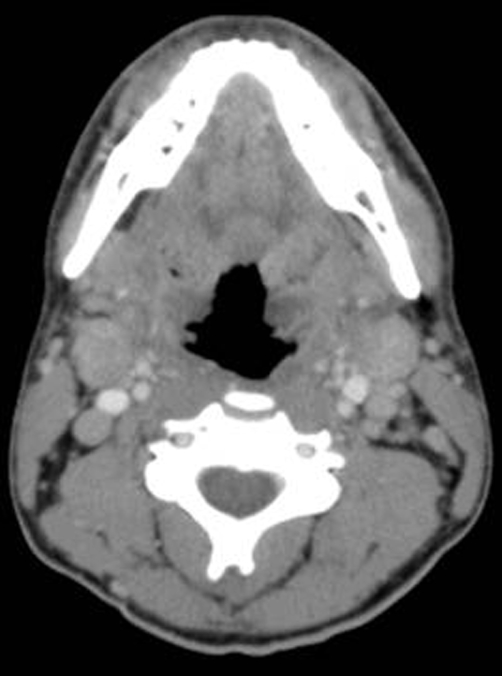
CT scan of head and neck. Note cervical lymphadenopathy.

In the emergency department, CT scan of the head and neck showed tonsillar swelling with lymphadenopathy (Figures 1 & 2). Laryngoscopy confirmed these findings and revealed no other causes of obstruction. He was treated with 10 mg intravenous dexamethasone and 3 g ampicillin/sulbactam with rapid improvement. The patient was then admitted to the medical service where steroid treatments were continued overnight and he was discharged the following day on no medications.

## Discussion

Airway compromise due to swelling of pharyngeal tissues can result from many infectious and non-infectious processes. While this patient already carried a diagnosis of infectious mononucleosis, we could rule out diphtheria as there were no significant pharyngeal exudates and he had been previously vaccinated. Direct visualization of the pharynx with laryngoscopy revealed no foreign bodies, neoplasms, abscesses, and revealed only tonsillar swelling. Cultures for superimposed streptococcus infection were negative. Taken together, these diagnostic findings further confirmed that this patient’s symptoms were due only to infectious mononucleosis without exacerbating processes.

Infectious mononucleosis is often an uncomplicated, self-limited illness resulting from Epstein-Barr Virus infection in 90% of cases and Cytomegalovirus in 5-10% of cases. Though it is mildly symptomatic in young children and elderly adults, young adults manifest a prodrome of fatigue and malaise followed by fever, lymphadenopathy, and pharyngitis [3]. While splenic rupture and meningoencephalitis are often considered the most severe and morbid complications, upper respiratory tract or nasopharynx obstruction can also be fatal [4]. Tonsillar enlargement occurs regularly in infectious mononucleosis due to inflammation of the lymphoid tissue in Waldeyer’s ring [3]. However, this inflammation rarely leads to airway compromise and hospital admission [5]. Obstructive symptoms often resolve with steroid treatment and rarely require intubation. In one study of 467 patients admitted to a hospital with infectious mononucleosis, only five patients presented with potentially lethal airway obstruction and only two of these individuals did not respond to medical therapy [6]. In such instances, the obstruction can be severe enough to warrant surgical intervention. In one published case report, two teenage patients with mononucleosis required tonsillectomy in order to breathe adequately [7].

While “hot potato voice” is used to describe vocal changes in those with pharyngeal pathology, it may not be accurate terminology. According to a study comparing vowel articulation between patients with peritonsillitis and healthy controls with an actual hot potato in their mouth, there was a significant difference in formant frequencies between the two groups [8]. The study concluded that the shape of the vocal tract is changed in peritonsillitis while tongue movements are dysfunctional in the presence of a hot potato with the resultant vocal changes being distinct between the two situations. Whether clinicians use the term correctly or not, a suspicion of “hot potato voice” on physical exam should be approached with appropriate airway management and investigation into the underlying etiology.

## Conclusion

The patient described in this report suffered from tonsillar swelling leading to airway compromise, a rare complication of infectious mononucleosis that can be fatal in some instances. However, no co-morbid infectious processes were identified. In this patient, a recently diagnosed mononucleosis allowed rapid characterization of the etiology of his dyspnea and dysphagia and thus prompt treatment with steroids. Airway compromise can develop rapidly and thus patients should be advised about this serious complication in addition to splenic rupture and meningitis.

## Abbreviations

AST: apartate aminotransferase
ALT: alanine aminotransferase
mg: milligram
g: gram
